# Isocitrate Dehydrogenase (IDH)-Mutant High-Grade Glioma in the Cerebellum: A Case Report

**DOI:** 10.7759/cureus.63335

**Published:** 2024-06-27

**Authors:** Divya Dinesh, Thanka Johnson, Divyalakshmi P.K., Santhanam Rengarajan, Venkatraman Indiran

**Affiliations:** 1 Pathology, Sree Balaji Medical College and Hospital, Chennai, IND; 2 Neurosurgery, Sree Balaji Medical College and Hospital, Chennai, IND; 3 Radiodiagnosis, Sree Balaji Medical College and Hospital, Chennai, IND

**Keywords:** intra axial lesion, supra tentorial, cerebellar, isocitrate dehydrogenase, high-grade glioma

## Abstract

High-grade glial cancers typically arise in the cerebral hemisphere and rarely in the cerebellum. Our objective was to highlight the diagnostic features of isocitrate dehydrogenase (IDH)-mutant high-grade gliomas in the cerebellum. We present a case of an elderly patient admitted with giddiness who was diagnosed with IDH-mutant high-grade glioma in the cerebellum, presenting as multiple lesions. We evaluated an open biopsy specimen to arrive at a diagnosis and used molecular studies to confirm the diagnosis and further categorize the specimens. Histopathology and immunohistochemistry confirmed the diagnosis of IDH-mutant high-grade glioma in the cerebellum.

## Introduction

Central nervous system (CNS) tumors account for only 2% of all malignant neoplasms. Nearly half of the tumors in the CNS are benign [1]. Gliomas are the most common primary malignant brain tumors in adults, presenting as supratentorial lesions. Cerebellar gliomas are rare and account for approximately 1% of CNS tumors. Cerebellar high-grade gliomas (cHGGs) have a poor prognosis and preferentially affect males and young adults [2].

## Case presentation

**Figure 1:**
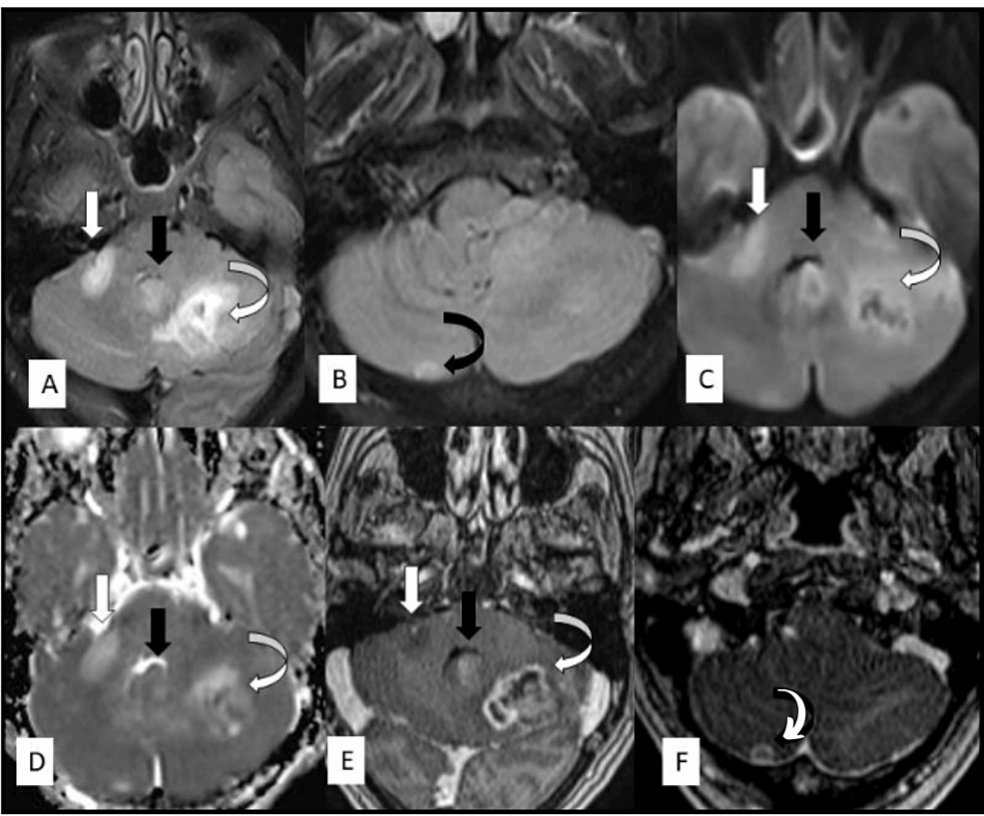
MRI axial T2 image (A) and T2 FLAIR image (B) show three T2 hyperintense lesions in right middle cerebellar peduncle (white arrow), vermis (white arrow), left cerebellar hemisphere (curved white arrow), and right cerebellar hemisphere (curved black arrow). Axial DWI (C) and ADC map (D) show restricted diffusion in the left cerebellar and vermis lesions. Axial T1 post-contrast images (E,F) show heterogenous enhancement with necrotic center in the left cerebellar lesion, peripheral enhancement in right cerebellar lesion, solid enhancement in the cerebellar vermis, and absent enhancement in the right middle cerebellar peduncle lesion. FLAIR: fluid-attenuated inversion recovery; DWI: diffusion-weighted imaging; ADC: apparent diffusion coefficient

A 62-year-old woman came to the neurology outpatient department with complaints of giddiness and vomiting of one-week duration. On evaluation, plain and contrast brain MRIs showed three T2 hyperintense intra-axial lesions in the posterior fossa (right middle cerebellar peduncle, vermis, and left cerebellar hemisphere). In addition, axial T1 postcontrast images showed heterogenous enhancement with a necrotic center in the left cerebellar lesion, solid enhancement in the cerebellar vermis, and absent enhancement in the right-middle cerebellar peduncle lesion (Figure 1).

**Figure 2:**
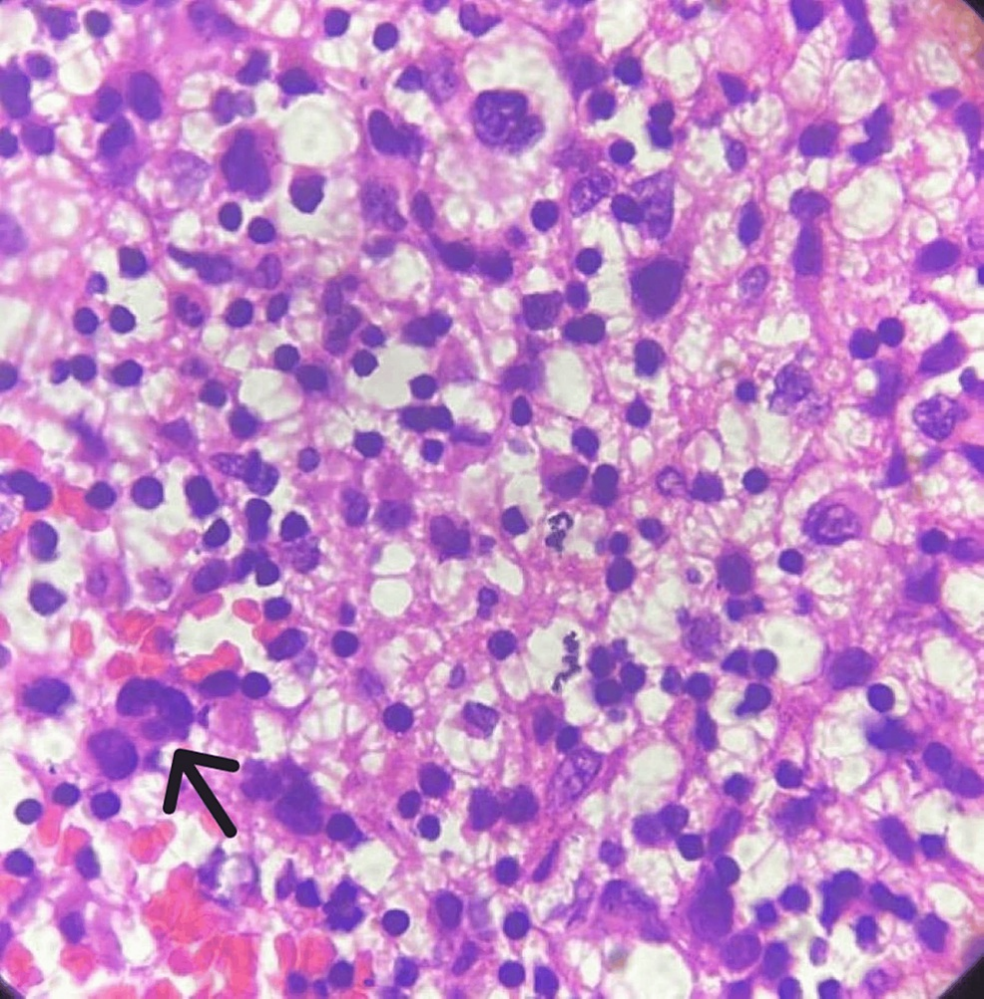
Photomicrograph of tumor cells showing eosinophilic cytoplasm with eccentrically placed nuclei. Black arrow shows a multinucleated cell (100x, H&E).

**Figure 3:**
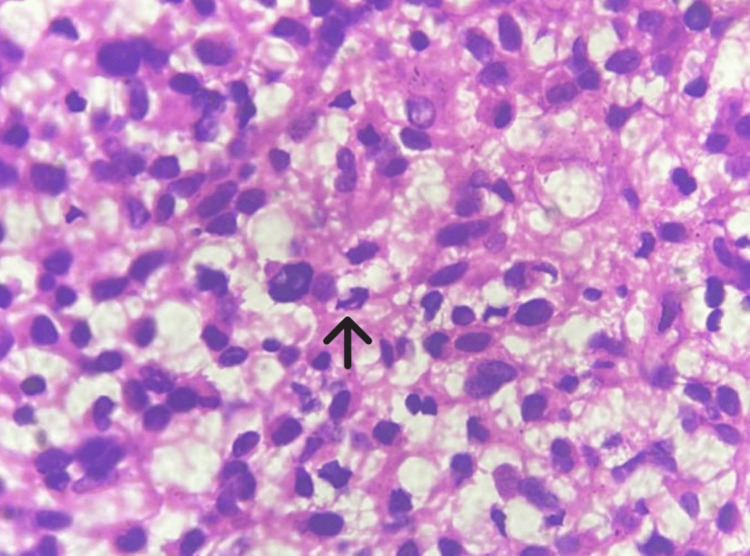
Photomicrograph showing mitosis (black arrow) (100x, H&E).

The patient underwent left-suboccipital decompression, and a biopsy was taken. Microscopy of the open biopsy specimen showed a glial tumor with pleomorphic cells having nuclear atypia, eccentrically placed nuclei, a moderate amount of glassy eosinophilic cytoplasm, and occasional binucleate and multinucleate cells (Figure 2), mitosis (Figure 3), and apoptosis. There was no necrosis, and microvascular proliferation was visible in the received specimen.

**Figure 4:**
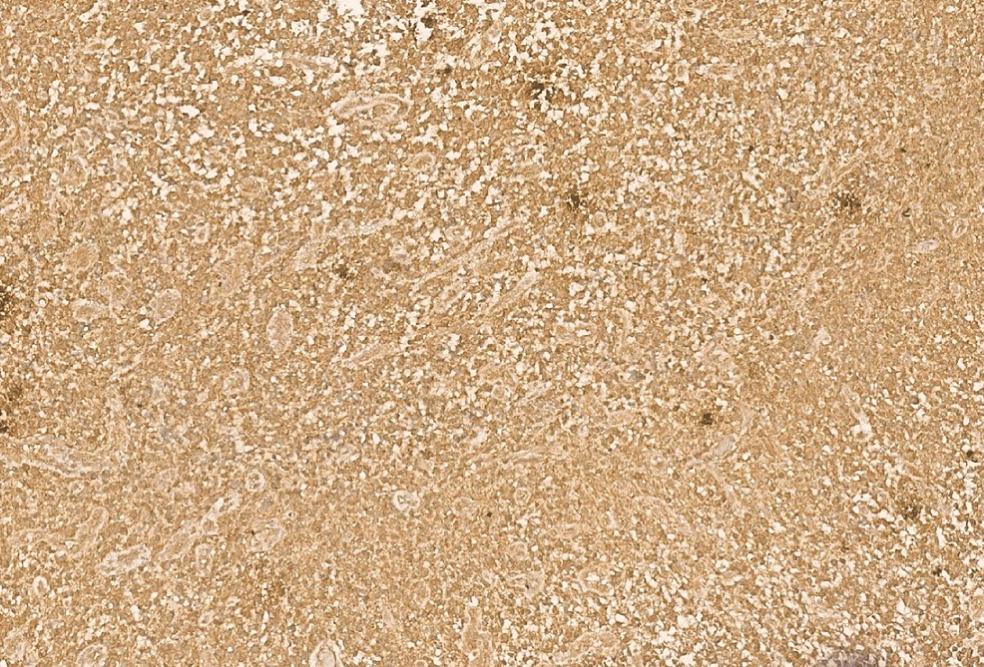
Photomicrograph showing IDH1 with diffuse cytoplasmic positivity (40x, H&E).

**Figure 5:**
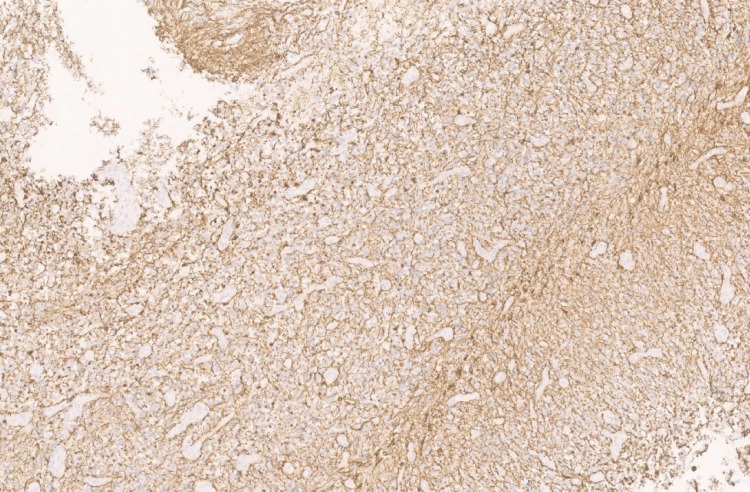
Photomicrograph showing GFAP with diffuse strong positivity (40x, H&E). GFAP: glial fibrillary acidic protein

**Figure 6:**
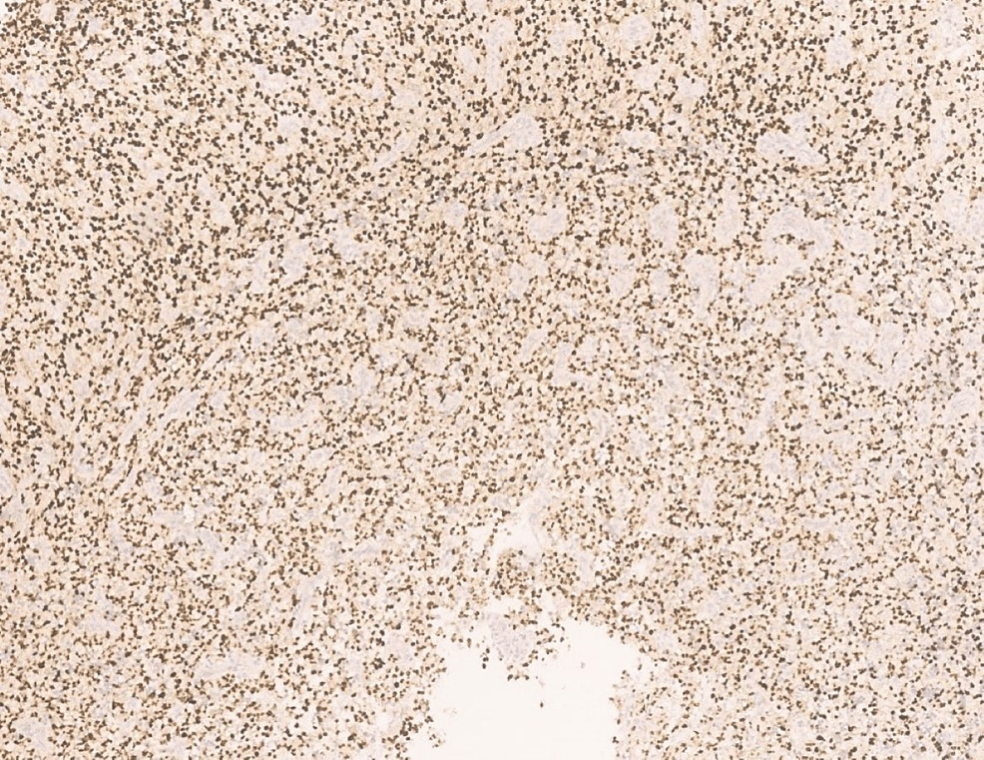
Photomicrograph showing Olig2 with diffuse nuclear positivity (40x, H&E).

Immunohistochemistry showed diffuse cytoplasmic positivity for isocitrate dehydrogenase (IDH) 1 (Figure 4), diffuse, strong positivity for glial fibrillary acidic protein (GFAP) (Figure 5), and diffuse nuclear positivity for Olig2 (Figure 6).

**Figure 7:**
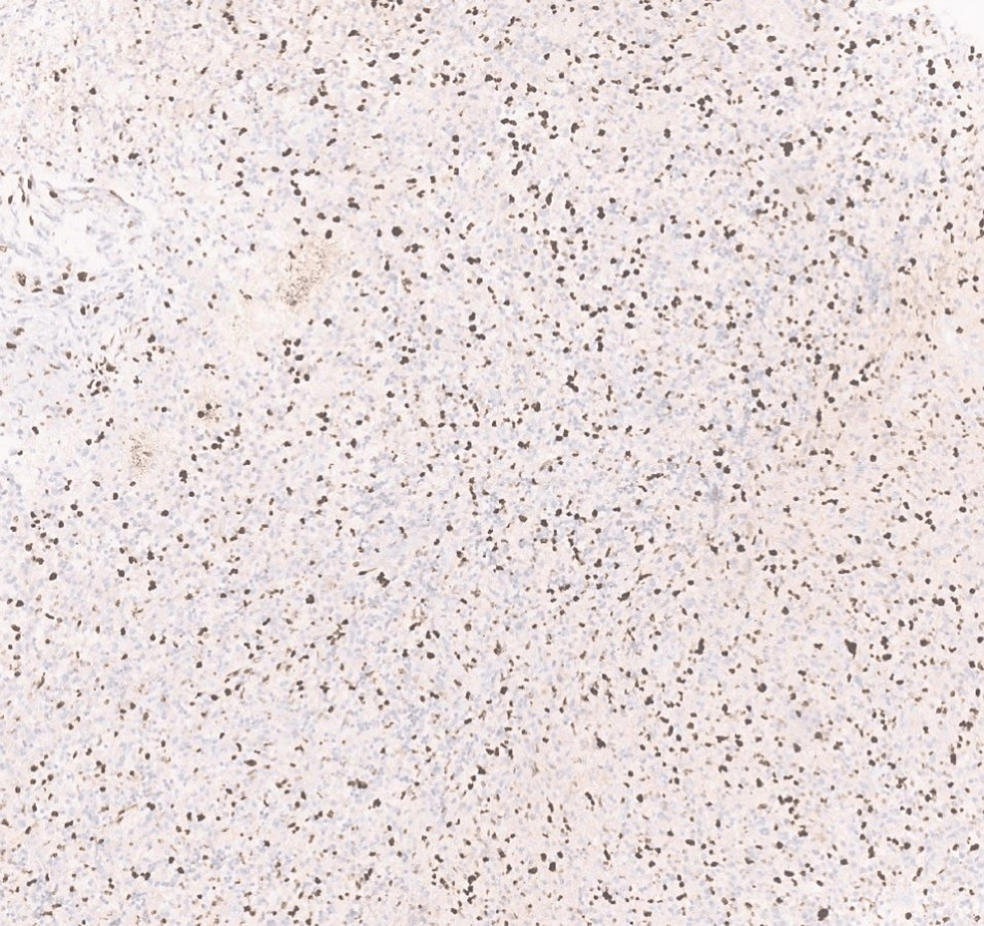
Photomicrograph showing Ki-67 with 40% positivity (40x, H&E).

**Figure 8:**
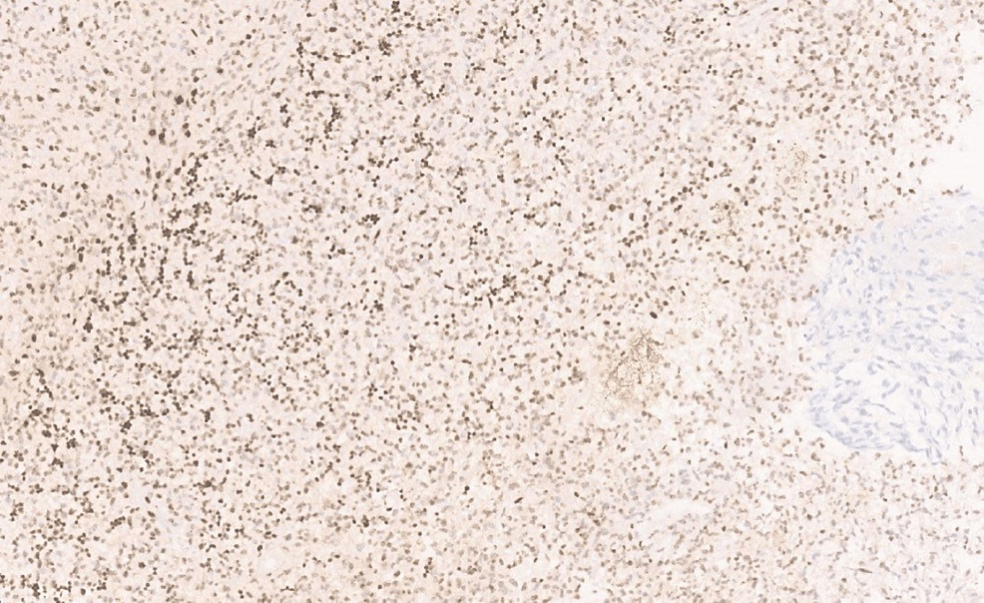
Photomicrograph showing H3K27m positivity in scattered cells (40x, H&E).

**Figure 9:**
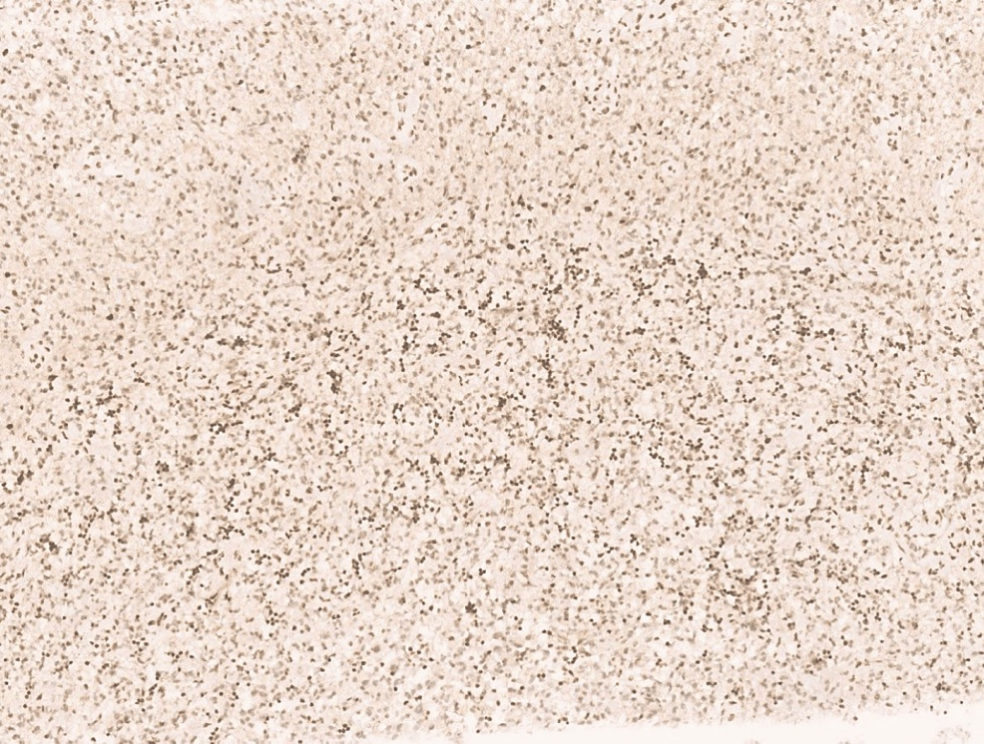
Photomicrograph showing ATRX retained in tumor cells (40x, H&E). ATRX: alpha-thalassemia/mental retardation, X-linked

Furthermore, Ki-67 showed 40% positivity (Figure 7), and p53 showed positivity in 10-15% of cells. There was scattered positivity for H3k27m (Figure 8), ATRX (alpha-thalassemia/mental retardation, X-linked) was retained in the cell (Figure 9), and epithelial membrane antigen (EMA) was negative.

After evaluating the histopathology and immunohistochemistry results, which showed highly pleomorphic cells with mitosis and IDH mutation respectively, we arrived at a diagnosis of IDH-mutant high-grade glioma in the cerebellum, and the patient was referred to another facility for further management. At present, the patient has completed radiotherapy and four cycles of chemotherapy with temozolomide.

## Discussion

Approximately 1% of high-grade gliomas in the CNS are cHGGs (WHO Grade 4) [2]. Subclasses of cHGG include high-grade astrocytomas with piloid features (HGAP) (~31% of tumors), H3k27m diffuse midline gliomas (~8% of tumors), and IDH wildtype glioblastomas (~43% of tumors). These tumor types were included in a recent series of cHGGs. In comparison to their supratentorial counterparts, gliomas occur disproportionately less frequently in the cerebellar parenchyma [3].

The actual presence of cHGGs was questioned until the 20th century because of their low incidence [3] and the possibly inadequate CT visibility of the posterior fossa structures [4]. The depletion of Substance P in the adult cerebellum is one theory that has been offered to explain this relative resistance to gliomagenesis in the cerebellum [5]. Some researchers have even suggested the majority of these cancers are metastases originating from an unknown supratentorial or brainstem location rather than the cerebellum [6]. Nevertheless, this theory is not well supported by the available data. The lack of FOX1-telencephalic markers and other hallmark gene expression, as well as other recent molecular findings, amply demonstrate that cHGGs are actually cerebellar in origin rather than cerebral.

The fifth edition of the WHO Classification of Head and Neck Tumors classifies adult-type gliomas into three types: (a) IDH-mutant astrocytomas presenting as diffusely infiltrating gliomas with IDH1 or IDH2 mutations and loss of nuclear ATRX expression, (b) oligodendrogliomas presenting with IDH mutations, combined whole arm deletions of 1p and 19q, and retained nuclear expression of ATRX, and (c) IDH-wildtype glioblastomas presenting with any one feature such as microvascular proliferation, necrosis, TERT (telomerase reverse transcriptase)-promoter mutations, epidermal growth factor receptor (EGFR) amplification, or +7/-10 chromosome copy number alterations [7].

Features of cHGGs include uncommon IDH mutations, rare EGFR amplification, moderately common p53 mutations [8], more frequent RAS (Rat sarcoma) mutations, ATRX alterations, PDGFRA (platelet-derived growth factor receptor alpha) amplification, less frequent TERT promotor mutations, CDK2a/2b loss, CDK4 amplification, scant expression of telencephalic markers (FOX1), widespread expression of cerebellar markers (PAX3) and widespread expression of oligodendrocyte progenitor markers (CSPG 4). Treatment for glioma is maximal resection followed by radiotherapy and concurrent chemotherapy with TMZ. Adjuvant TMZ or carmustine chemotherapy regimens are also often employed. Methylation classes of high-grade gliomas include (a) HGAPs, (b) diffuse midline gliomas H3k27-altered (DMG H3k27), (c) GBM IDH-wildtype subclass RTK I, and (d) GBM IDH-wildtype subclass midline (GBM-MID) [2].

HGAPs

A distinct DNA-methylation signature characterizes the recently identified tumor entity known as HGAP. These tumors most often develop in the posterior fossa, and the cerebellum is the most common site. Piloid astrocytomas typically occur in older patients. IDH wildtype HGAPs frequently exhibit CDK2a/2b deletions, ATRX mutations, and changes to the mitogen-activated protein kinase (MAPK) pathway. Potential treatments target the frequent MAPK pathway abnormalities in HGAP [9].

DMG H3k27m

An uncommon glioma known as DMG, H3k27-mutant, was mentioned in the fourth updated edition of the WHO Classification of Head and Neck Tumor classification of CNS malignancies. It was later renamed as DMG, H3 k27-altered in the fifth edition to reflect the presence of additional modifications such as EZHIP (Enhancer of Zeste Homologs Inhibitory Protein) overexpression [1,3]. Because this tumor typically develops in deep midline tissues such as the brainstem or thalamus and spreads widely, surgical removal is frequently not viable, and in some instances, even a biopsy is not an option. Neurosurgeons are also less likely to undertake resection or biopsy because of the poor prognosis [10].

IDH wildtype glioblastoma

Cerebellar glioblastomas are rare. These lesions are smaller and more frequently seen in young individuals [11]. IDH wildtype glioblastoma is an aggressive primary brain cancer with a median survival time of 14-21 months after diagnosis under ideal conditions. With the extended longevity of glioblastoma patients, extra neural glioblastoma metastasis is becoming more common, albeit still extremely rare. It is now expected that 2% of patients with glioblastoma may advance to extra neural metastasis, but this usually affects a small number of extra neural sites. Metastatic glioblastoma cases have been observed in the neck, lymph nodes, liver, lungs, bones, and systemic vasculature [12].

## Conclusions

Patients with high-grade glioma fall into two groups, those with a good prognosis and those with a poor prognosis. Younger patients often have a good prognosis when compared to older patients. The treatment regimen consists of maximum resection with adjuvant conventionally fractionated radiotherapy, with or without chemotherapy. However, the majority of high-grade glioma patients have a poor prognosis, and the most effective technique to treat them is currently unknown.

Given that cHGGs and supratentorial HGGs have distinct molecular characteristics, the cerebellum appears to be an area that is not prone to HGG formation. With the realization that a sizable fraction of these tumors may reflect HGAP or DMG H3k27m, the approach to treating cHGG is beginning to vary. In this report, the elderly female patient presenting with multiple lesions in the cerebellum had an atypical presentation.
